# Metabolic alterations and vulnerabilities in hepatocellular carcinoma

**DOI:** 10.1093/gastro/goaa066

**Published:** 2020-11-18

**Authors:** Daniel G Tenen, Li Chai, Justin L Tan

**Affiliations:** 1 Cancer Science Institute of Singapore, National University of Singapore, Singapore; 2 Harvard Stem Cell Institute, Harvard Medical School, Boston, MA, USA; 3 Department of Pathology, Brigham & Women's Hospital, Harvard Medical School, Boston, MA, USA; 4 Experimental Drug Development Centre, Agency for Science, Technology and Research (A*STAR), Singapore; 5 Genome Institute of Singapore, Agency for Science, Technology and Research (A*STAR), Singapore

**Keywords:** hepatocellular carcinoma (HCC), metabolism, metabolic vulnerability, targeted therapy

## Abstract

Liver cancer is a serious disease. It is ranked as the cancer with the second highest number of cancer-related deaths worldwide. Hepatocellular carcinoma (HCC), which arises from transformed hepatocytes, is the major subtype of liver cancer. It accounts for 85% of total liver-cancer cases. An important aspect of HCC that has been actively studied is its metabolism. With the liver as the primary site of numerous metabolic processes in the body, it has been shown that the metabolism of HCC cells is highly dysregulated compared to that of normal hepatocytes. It is therefore crucial to understand the metabolic alterations caused by HCC and the underlying mechanisms for these alterations. This deeper understanding will allow diagnostic and therapeutic advancements in the treatment of HCC. In this review, we will summarize the current literature in HCC metabolic alterations, induced vulnerabilities, and potential therapeutic interventions.

## Introduction

The liver is the site of major metabolic processes in the body, including detoxification of blood, production of bile for the breakdown of fats in the digestive track, the storage of glucose in the form of glycogen, and the synthesis of amino-acid precursors that make up proteins. Hepatocytes, which make up ∼85% of the total mass of the liver [1], are responsible for the majority of these metabolic processes. It is perhaps unsurprising that hepatocellular carcinoma (HCC)—the liver-cancer subtype that originates from the hepatocytes—results in the dysregulation of a large number of metabolic processes to fuel tumorigenesis [2–4]. Liver cancer is the second leading cause of cancer deaths worldwide [5]. HCC accounts for 85% of total liver-cancer cases and risk factors include chronic hepatitis B virus (HBV) and hepatitis C virus (HCV) infection [6], alcohol intake, diabetes, obesity [7, 8], and non-alcoholic fatty liver disease (NAFLD) [9]. While metabolic dysregulation has also been characterized in other primary liver-cancer subtypes such as cholangiocarcinoma and angiosarcoma [10], this review will primarily focus on metabolic alterations and induced vulnerabilities caused by these alterations in HCC.

In HCC, metabolic processes ranging from glucose metabolism and energy generation in the form of adenosine triphosphate (ATP) to amino-acid and fatty-acid metabolism are significantly altered in the disease. These alterations serve to augment the ability of the tumor to thrive, proliferate, and metastasis. However, the increased dependency of the tumor on some of these pathways can lead to metabolic vulnerabilities, which can be exploited by inhibitors of these pathways to therapeutically target HCC. Thus far, a significant number of metabolic pathways have been examined in detail in HCC to better understand their roles in tumorigenesis. However, there remain some processes, such as nucleotide metabolism, for which some preliminary evidence suggests a role in HCC, but detailed tumorigenic mechanisms have yet to be elucidated.

## Glycolysis and glycogen metabolism

**Figure 1.**
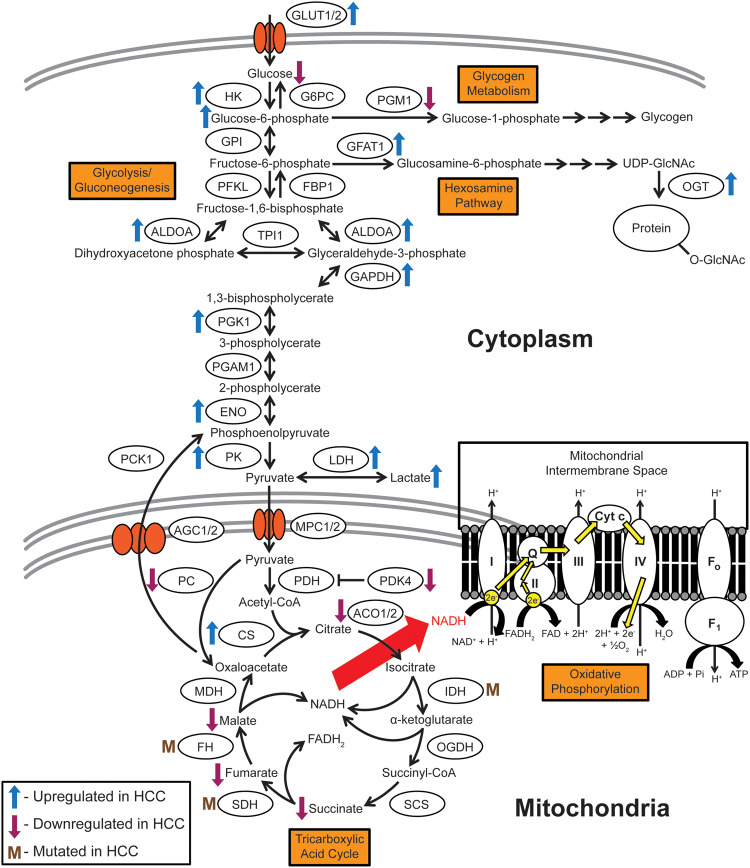
Schematic of glycolysis, gluconeogenesis, glycogen metabolism, the hexosamine pathway, oxidative phosphorylation, and the TCA cycle. Glycolysis and its opposing pathway, gluconeogenesis, serve as a central hub for the utilization of glucose in multiple pathways such as glycogen metabolism, the hexosamine pathway, the TCA cycle, and downstream oxidative phosphorylation. Tumors require energy to grow and proliferate, and the glycolysis–TCA cycle–oxidative phosphorylation axis provides significant amounts of cellular energy in the form of ATP. These pathways feature significantly in HCC and their dysregulation provides insight into tumorigenic mechanisms that can be potentially targeted with precision therapeutics. Metabolite nomenclature: ADP, adenosine diphosphate; ATP, adenosine triphosphate; CoA, coenzyme A; FAD, flavin adenine dinucleotide; FADH_2_, reduced flavin adenine dinucleotide; NAD, nicotinamide adenine dinucleotide. Enzyme nomenclature: I-IV, mitochondrial complexes I-IV; ACO1/2, aconitase 1/2; AGC1/2, aspartate/glutamate carrier 1/2; ALDOA: aldolase A, fructose bisphosphate; CS, citrate synthase; Cyt c, cytochrome c; ENO, enolase; F_0_, F_0_ subunit of ATP synthase (mitochondrial complex V); F_1_, F_1_ subunit of ATP synthase (mitochondrial complex V); FBP1, fructose bisphosphatase 1; FH, fumarate hydratase; G6PC, glucose-6-phosphatase; GAPDH, glyceraldehyde-3-phosphate dehydrogenase; GFAT1, glutamine: fructose-6-phosphate transaminase 1; GLUT1/2, glucose transporter type 1/2; GPI, glucose-6-phosphate isomerase; HK, hexokinase; IDH, isocitrate dehydrogenase; LDH, lactate dehydrogenase; MDH, malate dehydrogenase; MPC1/2, mitochondrial pyruvate carrier 1/2; OGDH, oxoglutarate dehydrogenase; OGT, O-linked N-acetylglucosamine (O-GlcNAc) transferase; PC, pyruvate carboxylase; PCK1, phosphoenolpyruvate carboxykinase 1; PDH, pyruvate dehydrogenase; PDK4, pyruvate dehydrogenase kinase 4; PFKL, phosphofructokinase liver type; PGAM1, phosphoglycerate mutase 1; PGM1, phosphoglucomutase 1; PGK1, phosphoglycerate kinase 1; Pi, inorganic phosphate; PK, pyruvate kinase; Q, coenzyme Q; SCS, succinyl coenzyme A synthetase; SDH, succinate dehydrogenase; TPI1, triosephosphate isomerase.

The liver plays a major role in glucose metabolism. It metabolizes glucose through glycolysis for energy but can also store excess glucose in the form of glycogen. The liver releases glucose to the rest of the body through the breakdown of glycogen stores (glycogenolysis) and generation of glucose from other proteins and lipids (gluconeogenesis) [11]. The dysregulation of and dependency on glucose metabolism is one of the earliest metabolic alterations described in cancer. Otto Warburg initially proposed that cancer cells favor glycolysis as a quick source of energy, in the form of ATP, for rapid growth and proliferation over the more energy-efficient oxidative phosphorylation pathway [12]. Glycolysis enzymes are primarily upregulated in HCC, while the glycogen metabolism enzyme phosphoglucomutase 1 (PGM1) is downregulated, suggesting a preference for glucose to undergo glycolysis instead of being stored as glycogen [2–4] (Figure 1).

Before glucose can be metabolized, it has to enter cells. Glucose transporters GLUT1 [13, 14] and GLUT2 [15, 16], which are critical for glucose uptake into cells, show increased levels in HCC (Figure 1). This upregulation might be related to oncogene MYC expression, since GLUT1 is transcriptionally upregulated in mouse models where Myc is overexpressed in the liver [17].

Once glucose is transported into the cytosol, hexokinase (HK) enzymes convert it into glucose-6-phosphate (G6P) as the first step in glycolysis (Figure 1). G6P levels are elevated in HCC compared to those in normal liver [18]. This might be due to hexokinase HK2 isoform upregulation in HCC, which contributes to an increased rate of G6P conversion from glucose [19–21]. Silencing HK2 in HCC cells suppresses their cell growth and proliferation in culture and *in vivo* [21]. The lack of HK2 activity upregulates oxidative phosphorylation, sensitizing HCC cells to the oxidative phosphorylation inhibitor metformin [21]. The synergistic effects of HK2 ablation and metformin in HCC cells suggest that the development of clinical hexokinase inhibitors in combination with oxidative phosphorylation inhibitors could potentially target these metabolic vulnerabilities successfully.

The next significantly altered glycolytic step in HCC is the conversion of phosphoenolpyruvate to pyruvate by the pyruvate kinase (PK) enzyme (Figure 1). The PKLR and PKM genes code for four PK splice isoforms: PKL, PKR, PKM1, and PKM2 [22–24]. PKL is expressed in normal liver [23]. PKM2, however, is upregulated in HCC, while PKM1 and PKL levels remain unchanged, and PKR is undetectable [25]. In mouse models, Myc induction lowers PKL levels [26]. High PKM2 expression correlates with poor prognosis in HCC patients [27, 28]. PKM2 also shows higher enzymatic activity in HCC cells compared to that in adjacent normal tissue [28]. On the contrary, murine PKM2 knockouts promote HCC [29], suggesting a more complicated mechanism for how PKM2 influences HCC tumorigenesis. Myc mouse tumors reflect an increase in PKM1/2 levels [26]. The interplay among PK isoforms in HCC remains unclear and should be further investigated.

In anaerobic respiration, pyruvate is converted into lactate instead of acetyl-coenzyme A (acetyl-CoA) that enters the tricarboxylic acid (TCA) cycle (Figure 1). This conversion is catalysed by lactate dehydrogenase (LDH). High levels of LDH observed in HCC patients simultaneously raises lactate levels [30] and is a risk factor for HCC recurrence [31]. Sorafenib-treated patients with high serum levels of LDH showed decreased progression-free survival [32]. Since the LDH A subunit (LDHA) is upregulated in a range of different cancers and LDHA-targeting therapeutics are available [33], it is important to study this gene’s impact on HCC in greater detail.

A number of factors have been shown to influence glycolysis and gluconeogenesis through the upstream gene regulation of metabolic enzymes. Transmembrane glycoprotein CD147 has been shown to upregulate glycolysis through p53-dependent upregulation of GLUT1 and PFKL, the liver-specific isoform of phosphofructokinase [34]. CD147 also downregulates mitochondrial biogenesis genes such as peroxisome proliferator-activated receptor gamma co-activator 1-alpha (PGC1α) and transcription factor A, mitochondrial, suggesting a reverse effect on mitochondrial energetic processes such as the TCA cycle and oxidative phosphorylation [34]. HCV infection in primary human hepatocytes upregulates glycolysis through the activation of transcription factor hepatocyte nuclear factor 4-alpha (HNF4α), which in turn transcriptionally upregulates glycolytic genes such as PKLR [35]. Interestingly, HCV infection in a HCC cell line has been shown to upregulate gluconeogenesis through the regulation of gluconeogenic transcription factors such as FoxO1 by histone deacetylase 9 (HDAC9) [36]. The upstream regulatory mechanisms of glucose metabolism gene regulation in HCC are not as well characterized and require greater understanding.

In terms of studies on drugging glucose metabolism to treat HCC, there have been some encouraging results. The administration of the diabetic drug metformin, which lowers the amount of sugar produced in the liver and sensitizes muscle cells to insulin, has been shown to decrease HCC risk [37] and is associated with reduced recurrence in increased overall HCC patient survival post hepatic resection [38]. In addition, a novel compound combining metformin and rosiglitazone, the latter a compound that blocks peroxisome proliferator-activated receptors in fat cells to make them more responsive to insulin, has been shown to suppress HCC [39]. With further research efforts, there is potential for the development of drugs targeting glucose metabolism in HCC.

## TCA cycle

The TCA cycle utilizes pyruvate from glycolysis to generate reduced nicotinamide adenine dinucleotide (NADH) and reduced flavin adenine dinucleotide (FADH_2_)—cofactors that channel electrons to oxidative phosphorylation for downstream energy generation (Figure 1). TCA metabolic intermediates such as succinate, fumarate, and malate are reduced in HCC [18].

In addition, TCA enzyme expression levels are also altered in HCC [2–4]. Pyruvate dehydrogenase (PDH) converts pyruvate from glycolysis into acetyl-CoA, which enters the TCA cycle (Figure 1). Downregulation of pyruvate dehydrogenase kinase 4 (PDK4), which inhibits PDH by phosphorylation, is associated with poor prognosis in HCC [40]. Interestingly, the knockout of PDK4 did not affect oxidative phosphorylation and glycolysis, but instead upregulated lipogenesis [40]. Succinate dehydrogenase (SDH), which converts succinate into fumarate, and fumarate hydratase (FH), which converts fumarate into malate (Figure 1), potentially function as tumor suppressors, since they tend to gain loss-of-function mutations [41, 42]. As a result, the build-up of succinate and fumarate stabilizes transcription factor hypoxia-inducible factor 1-alpha (HIF-1α), transcriptionally activating glycolysis and angiogenesis [42]. The SDH B isoform is decreased in HCC and this change is associated with tumorigenic phenotypes [43]. Mutations in isocitrate dehydrogenase (IDH), the enzyme that converts isocitrate into α-ketoglutarate (Figure 1), are rampant in multiple cancer types but are rarely observed (∼2%) in HCC [10]. The upstream regulatory mechanisms that alter TCA cycle enzyme expression and activity in HCC have yet to be thoroughly explored.

## Oxidative phosphorylation

Oxidative phosphorylation is the major energy-producing metabolic process in the cell, generating large amounts of ATP from NADH and FADH_2_ produced in the TCA cycle (Figure 1). Electrons from the oxidation of NADH and FADH_2_ are transferred through membrane-bound complexes in the mitochondrial inner membrane, with the energy derived utilized to activate proton pumps. These pumps create a potential gradient across the mitochondrial inner membrane by transporting positively charged hydrogen ions from the mitochondrial matrix into the intermembrane space. Oxidative phosphorylation is therefore also known as the electron-transport chain.

Impaired oxidative phosphorylation is associated with increased tumorigenicity in HCC [44, 45]. HCV infection downregulates oxidative phosphorylation-associated protein subunit expression, reminiscent of the Warburg effect [46]. Mitochondrial microRNAs have also been shown to downregulate oxidative phosphorylation gene expression in HCC, thereby promoting glucose metabolism [47]. Rab3A, a Ras-like GTPase important in membrane trafficking, can also dysregulate oxidative phosphorylation in HCC [48]. Rab3A is upregulated at both the transcript and protein levels in HCC tumor tissue [48]. Unlike in other cancers, where it acts as an oncogene, Rab3A inhibits HCC metastasis by enhancing oxidative phosphorylation, resulting in migration and invasion [48]. However, modification of Rab3a with N-acetylglucosamine (O-GlcNAc) attenuates these effects [48]. Since HCC tumors are commonly hyper O-GlcNAcylated, the tumor-suppressive function of Rab3A is likely diminished in HCC [48].

Upstream signaling and transcription factors have also been linked to modulating oxidative phosphorylation in HCC. Cytokine transforming growth factor beta (TGFβ), which induces migration and invasion in HCC, reduces oxidative phosphorylation with no alteration in glycolysis [45]. The stem-cell homeobox transcription factor NANOG is upregulated in alcohol and obesity-HCV-induced mouse models of HCC and in human tumor-initiating cells [49, 50]. NANOG in HCC tumor-initiating cells suppresses oxidative phosphorylation to support self-renewal and drug resistance [50]. Interestingly, as opposed to NANOG, stem-cell transcription factor spalt like transcription factor 4 (SALL4), which is upregulated in a subset of HCC, promotes oxidative phosphorylation through an increase in mitochondrial gene expression [51]. Mitochondrial oxidative phosphorylation inhibitors were shown to be particularly effective in suppressing SALL4-expressing HCC tumorigenesis in culture and *in vivo* [51]. The effectiveness of these mitochondrial inhibitors and aforementioned metformin, which both modulates glucose metabolism and inhibits oxidative phosphorylation, suggests the therapeutic potential of targeting oxidative phosphorylation in HCC patients.

## Hexosamine pathway

The hexosamine biosynthetic pathway (HBP) is required for the synthesis of uridine diphosphate N-acetylglucosamine (UDP-GlcNAc) for post-translational modification of proteins on their serine or threonine residues [52, 53] (Figure 1). This O-GlcNAc modification of proteins, catalysed by the O-GlcNAc transferase (OGT) enzyme, is a response to nutrient sensing and aberrant O-GlcNAcylation has been implicated in a number of diseases such as diabetes and cancer [54]. A high expression of glutamine: fructose-6-phosphate amidotransferase (GFAT1), the rate-limiting enzyme that converts fructose-6-phosphate into glucosamine-6-phosphate (Figure 1), correlates with poor HCC patient prognosis [55]. GFAT1 overexpression in HCC cell lines increases the tumorigenic phenotypes of proliferation and migration in culture [55]. Expression levels of the OGT enzyme are also increased in tumor tissue compared to those in non-cancerous controls [56]. Global O-GlcNAcylation levels are significantly increased in HCC tumors compared to those in healthy liver tissues, as well as in tumor tissues of HCC patients with recurring disease post liver transplantation [57].

In terms of mechanism, both OGT and the O-GlcNAc modification have been shown to influence the functionalities of various oncogenic proteins and processes. OGT upregulation has also been shown to regulate lipid metabolism and activate oncogenic pathways through increased oncogenic protein levels in HCC [58]. Increased O-GlcNAcylation leads to HCC tumorigenesis and metastasis through modification of oncogenic transcription factors such as c-Jun [59, 60]. O-GlcNAcylation of the receptor for activated C kinase 1 (RACK1) stabilizes it so that it can interact with the protein kinase C βII isoform, activating the kinase to phosphorylate eukaryotic translation initiation factor 4E (eIF4E) to translate oncogenes [61]. As mentioned in the oxidative phosphorylation section, tumor suppressor Rab3A is inactivated when modified with O-GlcNAc in HCC [48]. Both RACK1 and Rab3A O-GlcNAcylation promote HCC progression and metastasis [48, 61]. Interestingly, eIF4E itself can be O-GlcNAcylated, protecting it from proteasomal degradation, and subsequently promoting HCC cell-line proliferation and tumor-sphere formation [56]. More detailed studies of the effect of the HBP on the proteome in HCC could yield greater mechanistic and therapeutic insight into the disease.

## Pentose-phosphate pathway

**Figure 2.**
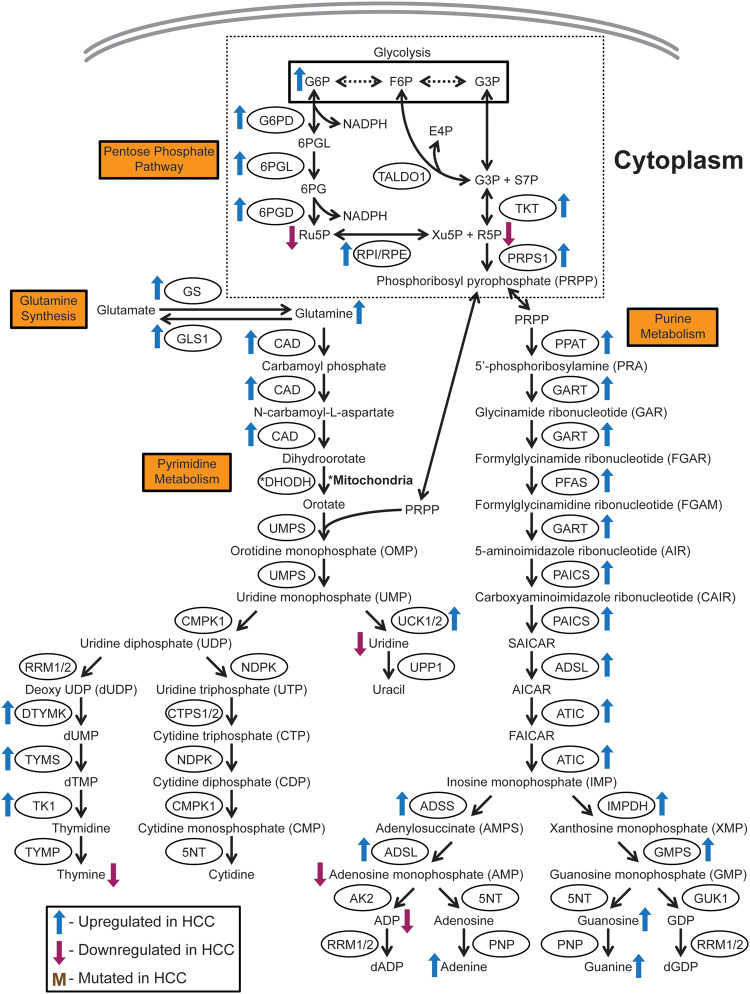
Schematic of glutamine synthesis, nucleotide biosynthesis, and the pentose-phosphate pathway. Nucleotide metabolism and its supporting processes such as the pentose-phosphate pathways and glutamine synthesis are significantly altered in HCC. Nucleotides are important precursors for DNA and RNA, as well as the energy currency ATP, which are needed for cell growth and proliferation. The exact mechanisms by which these pathways promote HCC tumorigenesis remain unclear however. Metabolite nomenclature: 6PG, 6-phosphogluconate; 6PGL, 6-phosphogluconolactone; ADP, adenosine diphosphate; AICAR, 5-aminoimidazole-4-carboxamide ribonucleotide; dADP, deoxyadenosine diphosphate; dGDP, deoxyguanosine diphosphate; dTMP, deoxythymidine monophosphate; dUMP, deoxyuridine monophosphate; E4P, erythrose-4-phosphate; F6P, fructose-6-phosphate; FAICAR, 5-formamidoimidazole-4-carboxamide ribonucleotide; G3P, glyceraldehyde-3-phosphate; G6P, glucose-6-phosphate; GDP, guanosine diphosphate; NADPH, reduced nicotinamide adenine dinucleotide phosphate; R5P, ribose-5-phosphate; Ru5P, ribulose-5-phospahte; S7P, sedoheptulose-7-phosphate; SAICAR, succinyl 5-aminoimidazole-4-carboxamide ribonucleotide; Xu5P, xylulose-5-phospahte. Enzyme nomenclature: 5NT, 5’-nucleotidase; 6PGD, 6-phosphogluconate dehydrogenase; 6PGL, 6-phosphogluconolactonase; ADSL, adenylosuccinate lyase; ADSS, adenylosuccinate synthetase; AK2, adenylate kinase 2; ATIC, 5-aminoimidazole-4-carboxamiade ribonucleotide formyltransferase/IMP cyclohydrolase; CAD, carbamoyl phosphate synthetase 2 aspartate transcarbamylase, and dihydroorotase; CMPK1, cytidine/uridine monophosphate kinase 1; CTPS1/2, cytidine triphosphate synthase 1/2; DHODH, dihydroorotate dehydrogenase; DTYML, deoxythymidylate kinase; G6PD, glucose-6-phosphate dehydrogenase; GART, glycinamide ribonucleotide synthetase, aminoimidazole ribonucleotide synthetase, and glycinamide ribonucleotide transformylase; GLS1, glutaminase 1; GMPS, guanine monophosphate synthase; GS, glutamine synthetase; GUK1, guanylate kinase 1; IMPDH, inosine monophosphate dehydrogenase; NDPK, nucleoside diphosphate kinase; PAICS, phosphoribosylaminoimidazole carboxylase and phosphoribosylaminoimidazole succinocarboxamide synthase; PFAS, phosphoribosylformylglycinamide synthase; PNP, purine nucleoside phosphorylase; PPAT, phosphoribosyl pyrophosphate amidotransferase; PRPS1, phosphoribosyl pyrophosphate synthetase 1; RPE, ribulose-5-phosphate-3-epimerase; RPI, ribose-5-phosphate isomerase; RRM1/2, ribonucleoside diphosphate reductase 1/2; TALDO1, transaldolase 1; TK1, thymidine kinase 1; TKT, transketolase; TYMP, thymidine phosphorylase; TYMS, thymidylate synthase; UCK1/2, uridine-cytidine kinase 1/2; UMPS, uridine monophosphate synthetase; UPP1, uridine phosphorylase 1.

The pentose-phosphate pathway (PPP) generates the ribose-5-phosphate backbone required for the synthesis of nucleotides, the precursors of DNA and RNA (Figure 2). The PPP also generates the cofactor-reduced nicotinamide adenine dinucleotide phosphate (NADPH) for lipid biosynthesis, maintaining glutathione and thioredoxin in reduced states, and for its antioxidant properties [62] (Figure 2). Nearly all PPP enzymes are transcriptionally upregulated [2–4], while PPP metabolites ribulose-5-phosphate (Ru5P) and ribose-5-phosphate (R5P) are downregulated [10, 18] in HCC. This suggests that the PPP is highly activated in HCC as metabolites are rapidly used up by the larger number of enzyme active sites available, possibly to feed precursor molecule phosphoribosyl pyrophosphate (PRPP) into nucleotide biosynthesis (Figure 2).

The conversion of G6P to 6-phosphogluconolactone (6PGL), catalysed by glucose-6-phosphate dehydrogenase (G6PD), is the first rate-limiting step of the PPP (Figure 2). Increased G6PD expression is associated with migration and invasion [63, 64], chemoresistance [65], metastasis, higher tumor grade, and decreased survival [66] in HCC. O-GlcNAcylation of G6PD induces tumorigenic activity, suggesting a synergistic interaction between the HBP and PPP in HCC [67]. HBV protein X (HBx) stimulates G6PD expression through oxidative stress transcription factor nuclear factor erythroid 2-related factor 2 (Nrf2) activation, implying a role for the PPP in HBV-induced HCC [66, 68].

Interestingly, Nrf2 overexpression itself has been shown to drive HCC [69]. Nrf2 also activates PPP enzyme transketolase (TKT), which converts glyceraldehyde-3-phosphate (G3P) and sedoheptulose-7-phosphate (S7P) into xylulose-5-phosphate (Xu5P) and R5P, in non-oxidative PPP to promote HCC by increasing antioxidants and purine biosynthesis, protecting cancer cells from reactive oxygen species toxicity [70]. In HCV-positive HCC, cell death and survival protein p62 phosphorylation prevents adaptor protein Kelch-like ECH-associated protein 1 (KEAP1) from recruiting the E3 ubiquitin ligase complex to ubiquitinate Nrf2, allowing active Nrf2 to activate the PPP [71]. A chemical inhibitor of KEAP1 that blocks its interaction with phosphorylated p62 facilitates Nrf2 degradation to suppress tumor-cell proliferation and increase sorafenib sensitivity in HCC cell lines [71]. The mechanisms by which the PPP promotes HCC have not been fully characterized and the fact that the PPP is crucial for NADPH generation and nucleotide biosynthesis suggests critical effects on downstream processes in tumorigenesis that should be examined in more detail.

## Nucleotide metabolism

Nucleotides form the building blocks of DNA and RNA molecules, as well as being used as the major energy currency in the cell in terms of ATP. Nucleotide biosynthesis requires R5P generated by the PPP and amino acids (Figure 2). Nucleotide metabolism genes involved in both purine and pyrimidine metabolism are transcriptionally upregulated in HCC [2–4, 72]. The levels of a number of nucleotides and nitrogenous bases are also altered [10, 18]. As mentioned previously, the PPP is also upregulated to supply metabolite PRPP that makes up the backbone of nucleotides. This suggests a greater demand for nucleotides by HCC, although the reasons for this are not well understood.

The carbamoyl phosphate synthetase 2, aspartate transcarbamylase, and dihydroorotase (CAD) gene, which codes for a multi-domain trifunctional enzyme that catalyses the first three steps of pyrimidine biosynthesis, is upregulated in HCC [72, 73]. The upregulation of a number of rate-limiting enzymes in pyrimidine biosynthesis such as deoxythymidylate kinase (DTYMK), thymidylate synthase (TYMS), and thymidine kinase 1 (TK1) in HCC is associated with cancer stemness and poor prognosis [3, 4, 74]. These three enzymes form a continuous cascade of reactions, in which DTYMK converts deoxyuridine diphosphate (dUDP) into deoxyuridine monophosphate (dUMP), TYMS converts dUMP into deoxythymidine monophosphate (dTMP), and TK1 converts dTMP into thymidine (Figure 2). Nearly all purine biosynthesis enzymes are upregulated in HCC [2–4]. Limited mechanistic insight is currently available on how nucleotide biosynthesis contributes to HCC. However, inhibiting nucleotide biosynthesis has shown therapeutic potential in acute myeloid leukemia and melanoma, suggesting that it is prudent to study this process in greater detail in HCC [75–77].

## Amino-acid metabolism

Amino acids are the building blocks of proteins and other important molecules such as heme, nucleotides, and neurotransmitters. A number of genes involved in amino-acid metabolism are significantly altered in HCC [2–4]. The amino acids glutamine and aspartate are upregulated while glycine is downregulated in HCC [10, 18].

Glutamine is a precursor for nucleotide biosynthesis. It is also used to replenish α-ketoglutarate, a key source of glutamate and glutamine for protein synthesis [78]. Lower plasma levels of glutamine have been observed in HCC patients due to increased uptake by tumors [18, 79]. The increased uptake can be explained by the upregulation of glutamine transporter ASCT2 in HCC cells [14]. Glutamine synthetase (GS) is the biosynthetic enzyme that converts glutamate into glutamine (Figure 2). GS is overexpressed and is a diagnostic marker for HCC [80]. HCC tumors in which Wnt signaling transducer β-catenin (CTNNB1) is mutated demonstrate higher GS expression, resulting in higher intracellular glutamine levels that activate the mammalian target of rapamycin complex 1 (mTORC1), a complex that activates protein translation for cell growth and proliferation [81]. This activation induces a vulnerability that can be exploited by mTORC1 inhibitors to target HCC [81]. In addition, a combination of asparaginase and GS inhibitor is able to hinder the growth of CTNNB1-mutated HCC cell-line xenografts [82]. TGFβ expression in HCC increases the levels of glutaminase 1 (GLS1), the enzyme that converts glutamine into glutamate (Figure 2), and upregulates the SLC7A5 glutamine transporter, in addition to its aforementioned effects on oxidative phosphorylation [45]. A possible reason for increased glutamate generation is for the anaplerotic conversion of glutamate to α-ketoglutarate to fuel the TCA cycle [45], although the significance of this is not well understood.

**Figure 3.**
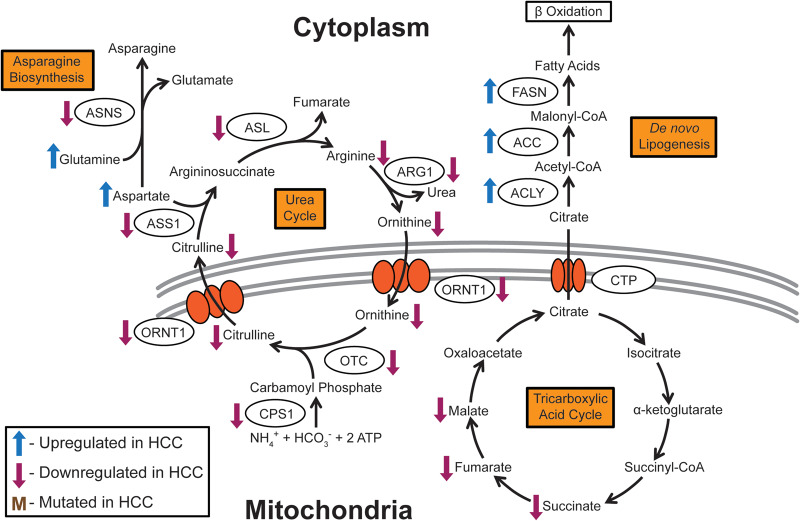
Schematic of asparagine biosynthesis, *de novo* lipogenesis, and the urea cycle. The urea cycle and fatty-acid biogenesis pathways, which are linked to important detoxification and lipid-metabolism functions in the liver, are significantly altered in HCC. However, the reasons why alterations in these pathways support tumorigenesis are not well defined. Enzyme nomenclature: ACC, acetyl-CoA carboxylase; ACLY, ATP citrate lyase; ARG1, arginase 1; ASL, argininosuccinate lyase; ASNS, asparagine synthetase; ASS1, argininosuccinate synthase 1; CPS1, carbamoyl phosphate synthetase 1; CTP, citrate transport protein; FASN, fatty-acid synthase; ORNT1, ornithine transporter 1; OTC, ornithine carbamoyltransferase.

The amino acid asparagine is essential for the formation of glycoproteins, as it serves as a site for sugar-group linkage. Asparagine synthetase (ASNS), which converts aspartate into asparagine (Figure 3), is expressed to a low extent in more malignant HCC [83]. ASNS re-expression can suppress tumorigenic phenotypes [83]. Low ASNS levels result in increased sensitivity to L-asparaginase treatment, which depletes asparagine in the cancer cells [83]. Asparaginase, as well as glutamine biosynthetic enzyme GS inhibitors, has been shown to arrest proliferation and induce apoptosis in HCC [84].

Methionine is the amino acid coded for by the initiation codon during translation and is therefore the first amino acid of polypeptides. Methionine metabolism occurs mainly in the liver. It has been shown that the knockout of S-adenosylmethionine synthase isoform type-1 (MAT1A), the enzyme that generates S-adenosylmethionine for the transfer of methyl groups in the cell, in mice causes HCC to develop [85]. The role of a small number of amino acids in HCC has been examined, with the functions of the majority of human amino acids in HCC having yet to be uncovered.

## Urea cycle

The liver is the major organ in which the urea cycle takes place. This process enables the body to excrete ammonia, generated from the breakdown of proteins, in the form of urea (Figure 3). Urea-cycle genes are predominantly decreased in HCC [2, 86]. In addition, lower levels of urea-cycle metabolites citrulline, arginine, and ornithine were observed in HCC patients compared to healthy individuals, and a decrease in levels is correlated with a later tumor stage [87]. Interestingly, urea-cycle metabolites are upregulated in the SALL4-expressing subset of HCC, but the reason for this remains unknown [51].

Urea-cycle rate-limiting enzyme carbamoyl phosphate synthetase 1 (CPS1) [73] and argininosuccinate synthase 1 (ASS1) [88] are hypermethylated in HCC, suggesting that they are epigenetically silenced. ASS1 downregulation is associated with cisplatin resistance [88]. CPS1 converts ammonia into carbamoyl phosphate, which enters the urea cycle, while ASS1 converts citrulline into argininosuccinate (Figure 3). Interestingly, arginine auxotrophy has been demonstrated in HCC as a result of ASS1 downregulation. This vulnerability can be exploited with PEGylated arginine deiminase treatment that depletes arginine levels by converting it into citrulline, starving HCC tumors [89, 90]. PEGylated arginase I, a second arginine-depleting recombinant enzyme, is also effective in targeting HCC [91]. However, Phase III clinical trials for arginine deiminase showed that, although it is well tolerated, it did not significantly increase overall survival [92]. More detailed mechanistic studies can shed light on how this downregulation of the urea cycle favors HCC tumorigenesis.

## Lipid metabolism

The liver synthesizes, stores, and breaks down lipids. It is therefore unsurprising that lipid metabolism is dysregulated in HCC to provide a steady source of lipids for membrane formation, energy generation, and post-translational modification to support tumorigenesis [93]. In HCC, fatty-acid biosynthesis genes such as fatty-acid synthase (FASN) and ATP citrate lyase (ACLY), cholesterol biosynthesis gene 3-hydroxy-3-methylglutaryl-CoA reductase (HMGCR), and adipogenesis transcriptional regulators such as sterol regulatory element-binding protein 1 (SREBP1) are upregulated [94]. FASN converts acetyl-CoA and malonyl-CoA into palmitate, ACLY converts citrate into acetyl-CoA (Figure 3), and HMGCR is a rate-limiting enzyme of the mevalonate pathway that converts 3-hydroxy-3-methylglutaryl-CoA into mevalonate for the generation of cholesterol and other isoprenoids. AKT/mTORC growth and proliferation signaling have been demonstrated to be responsible for these gene-expression changes as well as post-transcriptional modifications that increase lipogenesis to promote HCC [94]. Sphingolipids are also upregulated in the serum of HCC patients [95]. Alterations in the carnitine metabolism, which is essential for the transport of fatty acids into the mitochondria for energy generation through β-oxidation, are observed in chronic liver disease and HCC [96]. A high-fat and high-fructose diet in sedentary mice results in HCC [97, 98].

Lipid-metabolism enzymes have been shown to play a role in HCC. The acetyl-CoA carboxylase 1 (ACC1) and acetyl-CoA carboxylase 2 (ACC2) enzymes convert acetyl-CoA into malonyl-CoA, the major substrate for fatty-acid biosynthesis [99] (Figure 3). The knockout of ACC in mice enhances tumorigenesis in a diethylnitrosamine-induced-HCC model [100]. In addition, phospho-mutants of ACC1 and ACC2 result in NAFLD and HCC phenotypes in mice [101, 102]. Phosphorylation of ACC1 and ACC2 by adenosine monophosphate-activated protein kinase (AMPK) inhibits their enzyme activity [101]. FASN is upregulated in chemically and hormonally induced-HCC rat models [103]. However, overexpressing FASN in mouse models is not sufficient to induce tumorigenesis [104]. Mice with a knockout of acyl-CoA oxidase (AOX), which catalyses the first rate-determining step in peroxisomal fatty-acid β-oxidation, develop HCC [105]. This is due to prolonged activation of the transcription factor peroxisome proliferator-activated receptor alpha (PPARα) [105].

Upstream gene-regulatory factors also play a role in altering lipid metabolism in HCC. HBV transgenic mice show altered liver lipid metabolism [106]. Overexpression of HBV protein HBx induces lipid accumulation in cell lines and mouse liver [107]. This is explained by the concomitant increase in the expression levels of SREBP1 and peroxisome proliferator-activated receptor gamma (PPARγ), subsequently upregulating lipogenic and adipogenic enzymes [107]. HCV infection in primary human hepatocytes upregulates ketogenesis, the breakdown of fatty acids and ketogenic amino acids into ketone bodies, by activating transcription factors PPARα and retinoid X receptor (RXR) [35]. Mouse knockouts of Farnesoid X receptor (FXR), a nuclear receptor with essential roles in fatty-acid homeostasis, develop HCC [108]. In human HCC, pro-inflammatory cytokines are upregulated to inhibit transcription factor hepatocyte nuclear factor 1 alpha (HNF1α) activation of FXR, resulting in FXR inactivation reminiscence of the FXR knockout mouse model [109]. Stem-cell transcription factor NANOG activates fatty-acid oxidation in HCC-tumor-initiating cells to support self-renewal and drug resistance [50].

In terms of the role of cholesterol metabolism in HCC, the effect of HMGCR inhibitors, statins, has been examined in HCC. Statin use reduces the risk of HCC in the overall population [110], in chronic HBV-infected patients [111], decreases recurrence in HCC patients post tumor resection [112] and liver transplantation [113], and is associated with reduced mortality when administered post HCC diagnosis [114]. Future studies of the role of lipid metabolism in HCC could therefore yield additional therapeutic insight into the disease.

## Metabolic alterations induced by HCC risk factors

Besides understanding the metabolic alternations that occur in HCC, it is also important to consider the metabolic changes, if any, caused by the risk factors for HCC, such as chronic HBV and HCV infections, alcohol intake, diabetes, and NAFLD. This will give insight into whether the metabolic changes that occur during upstream HCC-initiation events are conserved when HCC develops and whether early intervention in suppressing these changes could slow down or prevent the development of HCC.

HBV induces metabolic alterations that are, for the most part, similar to alterations observed in HCC. HBV infection in host cells upregulates hexosamine and membrane phospholipid phosphatidylcholine biosynthesis through upregulating GFAT1 and choline kinase α (CHKA) [115]. Viral replication was shown to be dependent on the presence of these two metabolites, as inhibition of HBP and phosphatidylcholine biosynthesis significantly reduced HBV DNA levels [115]. This is in line with the previously highlighted observation that GFAT1 is upregulated in HCC patients with poor prognosis and that GFAT1 overexpression in cell-line models enhances tumorigenic phenotypes [55]. Similarly to those in HBV-infected cells, phosphatidylcholine levels are significantly upregulated in HCC patients as compared to liver cirrhotic patient controls [116].

HBV also alters lipid metabolism, which is also dysregulated in HCC, as discussed above (Figure 3). The expression of HBV viral proteins in mice alters liver lipid metabolism [106]. HBx overexpression induces lipid accumulation in cell culture and mouse models [107]. SREBP1 and PPARγ, as well as lipogenic and adipogenic enzymes, are upregulated [107]. This increase in lipid-metabolism gene expression is also observed in HCC [94].

Metabolomics performed on serum from HBV patients reveal viral hijacking of the glycerol-phosphate shuttle—the pathway that allows the reducing power of NADH generated by glycolysis to contribute to oxidative phosphorylation [117]. Glycolytic NADH is utilized by cytoplasmic glycerol-3-phosphate dehydrogenase to convert dihydroxyacetone phosphate (DHAP) into glycerol-3-phosphate. Glycerol-3-phosphate is subsequently reconverted into DHAP at the mitochondrial inner membrane via mitochondrial glycerol-3-phosphate dehydrogenase to generate FADH_2_ from FAD. FADH_2_ can then be utilized in oxidative phosphorylation (Figure 1). It is currently unknown whether the glycerol-phosphate shuttle is altered in HCC.

The progression of chronic HBV is also linked to an increase in long-chain triglycerides, citrulline, and ornithine from the urea cycle [117]. The increase in triglycerides is analogous to previous studies on the role of increased lipid metabolism in HCC as described. However, the observed increase in urea-cycle intermediates does not correlate with the decrease observed in HCC patients [87] (Figure 3). It remains unclear how the urea cycle factors into HCC pathogenesis and progression.

Similar to those of HBV, metabolic alterations induced by HCV also mirror the alterations observed in HCC. Glycolysis is significantly upregulated in HCC owing to the Warburg effect (Figure 1) and this upregulation is similarly observed in HCV infection in primary human hepatocytes, which upregulates glycolysis through the transcriptional upregulation of glycolytic genes [35]. HCV infection in a HCC cell line also upregulates gluconeogenesis, possibly to fuel energy generation through glycolysis in HCC [36]. HCV infection seems to support the Warburg effect by downregulating oxidative phosphorylation [46, 49] and this could explain the mechanism behind impaired oxidative phosphorylation in HCC [44, 45]. HCV infection in primary human hepatocytes activates lipid-metabolism transcription factor PPARα [35] and this activation is also observed in mouse knockouts of AOX, which develop HCC [105].

The effects of alcohol intake on liver metabolism have not been well characterized, but there is some association with metabolic alterations observed in HCC. CD36 is a gene that facilitates free-fatty-acid uptake and its expression is increased with chronic alcohol consumption [118]. CD36 knockout mice are resistant to liver steatosis when fed alcohol or a high-fat diet [119], implying that CD36 could play a role in lipid metabolism that increases the risk of HCC. Treatment of hepatoma cells with fatty acids and ethanol upregulates SREBP1c and PPARγ, and downregulates SIRT1, leading to impaired fatty-acid oxidation [120]. This is analogous to the upregulation of SREBP1 and PPARγ observed in HCC and HBV infection [94, 107]. It has also been observed that NAD+ levels are reduced with high blood-alcohol levels during binge drinking [121], although the direct consequence of this on HCC induction is unknown.

NAFLD is a HCC risk factor that exhibits lipid-metabolism dysregulation as a key feature since excessive lipid accumulation in the liver, in people with low or no alcohol consumption, is a hallmark of the disease [122, 123]. Fifty-nine percent of NAFLD patients who had biopsies for evaluation demonstrated progression to non-alcoholic steatohepatitis (NASH), owing to the onset of hepatocellular injury and inflammation [124–126]. As previously discussed, a mouse model for NASH fed with a high-fat diet, which leads to the development of steatohepatitis and eventually HCC, showed increased expression of lipid metabolism and insulin-signaling genes in the liver [97]. In HBP, O-GlcNAcylation of Rab3a has been linked to NAFLD-associated HCC since it regulates lipid metabolism [58]. This observation is complemented by the demonstration that Rab3A O-GlcNAcylation promotes HCC progression and metastasis [48].

Other than lipid metabolism, NAFLD also manifests alterations in other metabolic pathways that are reminiscent of HCC metabolism. NAFLD is prevalent among type 2 diabetes patients [127] and is associated with insulin resistance [128, 129]. As discussed, metformin reduces HCC risk and progression so this suggests a link between NAFLD and HCC. Branched-chain amino acids (BCAAs), leucine, isoleucine, and valine are elevated in NAFLD patient blood but the underlying mechanisms remain unknown [130]. Since BCAAs are known to regulate mTOR signaling, one postulation is that the observed elevation of these amino acids alters glucose metabolism [131–133]. While alterations in BCAA metabolism have not yet been reported in HCC, there have been reports of utilizing these amino acids to prevent and treat HCC [134]. Further studies could yield novel insight into how BCAAs play a role in the manifestation and progression of HCC.

NAFLD could also mirror HCC metabolism in terms of mitochondrial metabolism. In NASH patients, mitochondrial abnormalities have been observed [135]. NAFLD patients given ^2^H and ^13^C tracers to measure metabolites showed higher rates of lipolysis, gluconeogenesis, anaplerosis, and mitochondrial oxidative metabolism [129]. Alterations in these processes are mostly analogous to what has been observed in HCC. However, there is a need to better understand the underlying mechanisms governing these metabolic changes.

## Conclusion

Numerous metabolic processes feature heavily in promoting and supporting HCC tumorigenesis and metastasis. The majority of the major cellular metabolic pathways have been studied and validated to various extents in terms of their roles in HCC. From the current literature, HCC cells predominantly seem to demonstrate the Warburg effect. These cells prefer quick energy generation from glycolysis, through the conversion of glucose to lactate, instead of allowing pyruvate to enter the TCA cycle (Figure 1). As expected, the TCA cycle is downregulated in HCC, promoting the conversion of pyruvate into lactate (Figure 1). Interestingly, the downregulation in the TCA cycle also seems to promote flux of glycolytic metabolites through the PPP to supply precursors for nucleotide metabolism (Figure 2). The observed increase in nucleotide metabolism could provide the DNA and RNA precursors necessary for tumor-cell growth and proliferation, but this hypothesis has yet to be validated. In the case of oxidative phosphorylation, however, there exist genetic subsets of HCC in which this process is either upregulated as an energy source for tumorigenesis or downregulated in favor of glycolysis. The urea cycle, which is the excretion pathway for the byproducts of protein degradation, is downregulated in HCC, but the reasons for this remain unclear (Figure 3). There is also evidence for the upregulation of lipid metabolism (Figure 3) and the biosynthesis of some amino acids to provide energy and precursors that support tumorigenesis. Despite the wealth of information thus far, more has to be done to fully comprehend the range of metabolic alterations responsible for promoting HCC tumorigenesis.

Many of these metabolic processes have been studied in isolation, but it is likely that the cross talk between pathways and synergy among pathways plays an important role in HCC. Examples include the dual roles of TGFβ in reducing oxidative phosphorylation while enhancing glutamine anaplerosis [45], NANOG in repressing oxidative phosphorylation while upregulating fatty-acid oxidation in HCC tumor-initiating cells [50], and SALL4 in upregulating oxidative phosphorylation and urea-cycle intermediates [51]. A more complete understanding of metabolic alterations in HCC will enable a precision-medicine approach, in which patients with HCC metabolic subtypes can be diagnosed and treated with drugs targeting the metabolic vulnerabilities of these subtypes.

The characterization and validation of multiple metabolic vulnerabilities in HCC can also inform the use of combination therapy with metabolic inhibitors, which might elicit better outcomes for patients in the future. There are already a number of small molecules, such as inhibitors of oxidative phosphorylation, GS, and HMG-CoA reductase, available for targeting specific enzymes in the aforementioned metabolic pathways. Based on current and future studies of HCC metabolism, one can envision the future clinical development of combinations of metabolic drugs that can hopefully effectively treat HCC.

An interesting aspect of HCC metabolism that remains to be fully elucidated is HCC risk-factor-induced metabolic changes. There is evidence that risk factors such as HBV and HCV infections alter the metabolism of liver cells in a similar manner to that observed in HCC. However, there remain unanswered questions on how metabolic changes induced by alcohol intake, diabetes, and NAFLD relate to HCC. Having a complete understanding of how these pre-HCC metabolic changes precondition or encourage cells to progress to HCC might unlock new therapeutic strategies to slow or prevent the progression of these high-risk disease states to HCC. Overall, the study of the HCC metabolism in its entirety is timely and crucial, and will potentially serve as the basis for the development of better HCC therapeutic strategies in the long run.

## Funding

This work was supported by the Genome Institute of Singapore Innovation Fellow Award to J.L.T.; the Agency for Science, Technology, and Research A*ccelerate Gap Award [ETPL/18-GAP018-R20H] to J.L.T.; the Singapore Ministry of Health's National Medical Research Council Singapore Translational Research (STaR) Investigator Award to D.G.T.; the Singapore Ministry of Education under its Research Centres of Excellence initiative to D.G.T.; the National Institutes of Health [R35CA197697, P01HL131477] to D.G.T.; and the National Heart, Lung, and Blood Institute at the National Institutes of Health and Xiu Research Fund [P01HL095489] to L.C.

## Conflicts of interest

All authors declare no conflict of interest.

## Authors’ contribution and Acknowledgments

J.L.T. wrote the manuscript in consultation with D.G.T. and L.C. All authors read and confirmed this paper.
